# Cerebellar Contributions to Major Depression

**DOI:** 10.3389/fpsyt.2018.00634

**Published:** 2018-11-29

**Authors:** Malte S. Depping, Mike M. Schmitgen, Katharina M. Kubera, Robert C. Wolf

**Affiliations:** Department of General Psychiatry, Center for Psychosocial Medicine, University of Heidelberg, Heidelberg, Germany

**Keywords:** major depression, cerebellum, cerebro-cerebellar networks, VBM, intrinsic connectivity

## Abstract

Extending beyond the motor domain, the cerebellum is involved in various aspects of cognition and affect. Multidisciplinary evidence has demonstrated topographic organization of higher-order cognitive functions within the cerebellum. We here review recent neuroimaging research that indicates cerebellar contributions to major depressive disorder (MDD). At the structural level, increased volume of lobule IX has been demonstrated in MDD patients, independent of acute or remitted disease state. Successful treatment with electroconvulsive therapy has been associated with increased lobule VIIA volume in depressed patients. At the functional level, connectivity analyses have shown reduced cerebro-cerebellar coupling of lobules VI and VIIA/B with prefrontal, posterior parietal, and limbic regions in patients with MDD. As a limitation, most of this evidence is based on smaller patient samples with incomplete phenotypic and neuropsychological characterization and with heterogenous medication. Some studies did not apply cerebellum-optimized data analysis protocols. Taken together, MDD pathophysiology affects distinct subregions of the cerebellum that communicate with cortical networks subserving cognitive and self-referential processing. This mini-review synthesizes research evidence from cerebellar structural and functional neuroimaging in depression, and provides future perspectives for neuroimaging of cerebellar contributions to MDD.

## Introduction

Lesions of the cerebellar posterior hemispheres and the cerebellar vermis frequently result in cognitive and/or affective symptoms, sometimes referred to as the “cerebellar cognitive affective syndrome” (1). It is characterized by deficits in executive function and linguistic processing, as well as by emotion dysregulation (emotional lability, impaired social cognition, apathy, or depressed mood) (2). Electrical stimulation experiments in animal models have additionally related cerebellar neural activity to anxious and impulsive behavior (3–5). Over the past decade, cerebellar contributions to non-motor functions have attracted increasing scientific interest. In particular, structural and functional neuroimaging has allowed for a more detailed understanding of the “cognitive/affective cerebellum” (6, 7). Against this background, the question has been raised whether abnormal cerebellar structure or function may contribute to major depressive disorder (MDD) (8).

In this mini-review, we briefly summarize the available structural and functional neuroimaging data in humans regarding the role of the cerebellum in depression. We identify current research limitations and discuss future perspectives for neuroimaging of cerebellar contributions to MDD. To describe cerebellar anatomy, we use a revised version of the Larsell nomenclature (9), as suggested by Schmahmann et al. (10).

## Functional neuroanatomy of the “cognitive-affective cerebellum”

**Figure 1:**
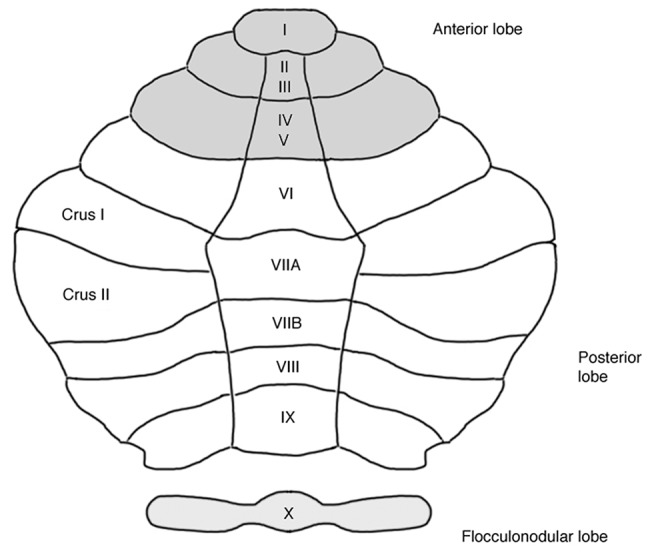
Unfolded view of the cerebellar cortex showing lobes and lobules **(I–X)**. Anatomical labeling according to a revised version of the Larsell nomenclature (9), as described by Schmahmann et al. (10). Each cerebellar lobule comprises an unpaired medial (i.e., vermal) portion and a bilateral hemispheric portion. The posterior lobe represents the “cognitive/affective” cerebellum. Approximately half of the cerebellar cortex is associated with non-motor functions (17), see text for details.

The cerebellum's histology is uniform throughout cortex, i.e., unlike the cerebral cortex, it has no discernable areal boundaries (11). The spatial organization of somatosensory, cognitive and affective representations within the cerebellum relies on polysynaptic connections that different subregions of the cerebellum form with functionally distinct regions of the cerebrum (12). MRI-Analyses of intrinsic functional connectivity (fcMRI) provide a means to discern theses cerebro-cerebellar circuits, thereby revealing the functional topography of the cerebellum. This evidence is corroborated by older electrophysiological and tract tracing experiments in animal models (13, 14). In healthy human subjects, several fcMRI studies of cerebro-cerebellar coupling are available (15–18). So far, the most comprehensive cerebellar mapping has been performed by Buckner et al. (17). Including 1,000 healthy subjects and employing different fcMRI approaches, the authors demonstrated that approximately half of the cerebellar cortex is associated with higher-level cognitive and affective functions (17). Non-motor functions are represented in the posterior lobe of the cerebellum, i.e., within cerebellar lobules VI–IX. Figure 1 shows an illustration of the unfolded cerebellar cortex. The intrinsic connectivity networks (ICN) (19) in which the non-motor cerebellum participates, include the “cognitive control network” (CCN) (20), the “salience network” (SN) (20), and the “default mode network” (DMN) (21). Additionally, a “cerebello-amygdaloid network” (CAN) has been suggested (18). Consistent evidence for cerebellar functional topography has emerged, especially within the cognitive domain. All available MRI studies of cerebellar connectivity in healthy subjects suggest that the anterior part of lobule VIIA, crus I and II, is related to the CCN (15–17, 22, 23). Two studies suggest that major connections to the DMN originate from the posterior part of crus I and II of lobule VIIA (15, 17); one study suggests that lobule IX is an essential cerebellar representation of the DMN (22). Cerebellar contributions to the SN seem to come from lobule VI (18, 23) and from lobules VIIB and VIII (23). Finally, lobules VI and VIII (18, 23), as well as the vermal portions of lobules VIIB, VIII, and IX (23) seem to have functional connections to the amygdala, potentially representing an independent ICN (18). In summary, there are functional subregions within, and sometimes extending beyond, each of the lobules VI–IX. Within one cerebellar lobule, segregated subregions are associated with distinct functional networks, differentially supporting cognitive or affective processing. An open research question is whether the hemispheric and the vermal portions of one lobule have the same (17) or separate (23) connectivity patterns.

Task-related cerebellar activations have been investigated in several MRI and PET studies. Verbal working memory, executive functions and emotional functions have been probed. These studies build on a cumulative number of ~100–150 subjects per task category. Stoodley and Schmahmann (6) conducted an “activation likelihood estimate” (ALE) meta-analysis of these findings. Consistent with the fcMRI studies, lobule VIIA, crus I was associated with cognitive functions (6). Further cognitive representations were detected in lobules VI and VIIB. The substrates of emotional processing were localized in lobule VI and in the vermal portion of lobule VII, in agreement with the functional connectivity results by Sang et al. (23) and Habas (18). Additional affective representations were localized in lobule VIIA, crus I (6).

In summary, the topographic organization of cognitive and affective representations in the cerebellum is complex. The cerebellar lobules within the “cognitive/affective cerebellum” consist of subregions with distinct connectivity patterns and functions. Across different imaging methodologies, there is most consistent evidence for the involvement of the anterior part of lobule VIIA, crus I and II, in cognitive processing (6, 15–18, 23). Clearly, there are also cerebellar contributions to the DMN, likely involving the posterior part of lobule VIIA, crus I and II (15, 17, 23), and potentially also lobule IX (15, 23). Furthermore, consistent evidence suggests that that lobule VI is associated with emotional processing (6, 18, 23). Finally, there is preliminary evidence for lobule VI involvement in the salience network (22, 23). Convergent findings are summarized in Table 1.

**Table 1:** Topographic organization of the non-motor cerebellum, as suggested by cerebellar neuroimaging.

**Psychological domain**	**Cerebellar subregion**	**Neuroimaging study**
Cognitive representations (CCN)	Lobule VIIA, crus I and II (anterior part)	(6, 15–17, 22, 23)
Self-referential representations (DMN)	Lobule VIIA, crus I and II (posterior part)	(15, 17, 23)
	Lobule VIIA (vermal part)	(17)
	Lobule IX	(15, 23)
Affective representations (CAN)	Lobule VI	(6, 18, 23)
Representations of the salience network (SN)	Lobule VI	(22, 23)

*Within one cerebellar lobule, segregated subregions are associated with distinct functional networks, differentially supporting cognitive, self-referential, affective, or salience processing, see text for details. CCN, cognitive control network; DMN, default mode-network; CAN, cerebello-amygdaloid network*.

## Abnormal cerebellar structure and function in patients with depression

Structural and functional changes of non-motor cerebellar regions in patients with depression have been subject to several MRI studies: Cerebellar connectivity has been investigated by six studies (24–29), cerebellar resting-state blood flow has been studied by one study (30), and cerebellum-optimized structural data analysis have been applied by two investigations (31, 32). Task-based imaging studies of the cerebellum in depressed patients are lacking at present.

### Cognitive representations

Consistent findings indicate that lobule VIIA, crus I and II, shows reduced connectivity to the cerebral components of the CCN in patients with depression, particularly to the dorsolateral prefrontal cortex (dlPFC) (24, 25, 27), but also to the ventromedial prefrontal cortex (vmPFC) (24, 28). In further support for the pathophysiological relevance of abnormal cerebellar-CCN coupling in depression, reduced lobule VIIA-vmPFC connectivity has been found to significantly correlate with impaired verbal working memory performance in depressed individuals (24). Finally, treatment with electroconvulsive therapy (ECT) seems to modulate the structure of an anterior subregion within lobule VIIA, crus I, in patients with severe acute depression (32). Notably, this structural change was associated with the antidepressant efficacy of ECT (32).

### Self-referential representations (DMN)

Aberrant activity of the DMN's cerebellar components has been repeatedly demonstrated in patients with depression (24, 25, 27, 28), however, the specific spatial distribution and the direction of change is unclear at present. In depressed patients, Guo et al. (27) reported reduced connectivity of lobule VII, crus I, to the inferior parietal cortex and to the inferior temporal cortex, Liu et al. (25) reported reduced connectivity of lobule VIIA, crus II, to the posterior cingulate cortex (PCC), while Alalade et al. (24) reported increased connectivity of the vermal portion of lobule VIIA to the PCC. The latter finding was associated with the severity of depressive symptoms (24). Aberrant functional connectivity of the cerebellar vermis with key components of the DMN, i.e., with the PCC (25), as well as with the anterior cingulate cortex and the vmPFC (28) have also been described. However, it is important to note that these studies did not consider the functional segmentation of the vermis. Taking into consideration that vermal topography is highly complex (7, 17, 23), only limited conclusions can be drawn from these investigations. In patients with depression, more sophisticated approaches are required to specify aberrant cerebellar DMN activity. Finally, preliminary evidence has been presented for structural abnormalities of a cerebellar DMN component in depression. Independent of acute or remitted disease stage, lobule IX volume appears to be increased in patients with recurrent depression (31).

### Representations of other domains

In depressed patients, no studies have yet been published on the structure or connectivity of cerebellar affective representations or of cerebellar representations of the salience network.

## The cerebellum in depression-state of knowledge and future perspectives

There is a special role for lobule VII within the “affective/cognitive cerebellum.” Unlike all other cerebellar lobules, lobule VII is not connected with the somatomotor system. Lobule VII communicates exclusively with cognitive and affective cerebral association cortices and with (para-) limbic structures (17). Cerebellar lobule VII contains subregions that are associated with cognitive, self-referential, and affective processing. In particular, strong evidence has accumulated for the involvement of lobule VII in cognitive processes. Convincing evidence has been provided for abnormal structure and function of lobule VII in patients with MDD. Multiple studies report decreased connectivity of lobule VII with cortical components of the CCN (24, 25, 27, 28). Preliminary findings also highlight the neuropsychological relevance of abnormal lobule VII-CCN coupling in patients with depression, i.e., an association between lobule VII-CCN connectivity and delayed memory recall (24). There are also cerebellar representations of the DMN and of the SN, as well as a putative cerebro-cerebellar ICN involving the amygdala (18), but their functional neuroanatomy is incompletely characterized at present.

There are two major limitations in current imaging approaches toward understanding the role of the cerebellum in depression. First, due to methodological constraints, the complex functional topography of the cerebellar vermis has been insufficiently addressed. At present, there is a considerable dearth of knowledge on dysfunction of the cerebellar vermis in depression. This is in contrast to the significance of the vermis for affective processes as illustrated by lesion studies (1). To accurately map the vermis' functional topography in depression, cerebellum-optimized data analysis protocols should be used by future MRI investigations. Cerebellum-optimized protocols have proven to result in superior neuroanatomical precision compared to conventional methods (33). Second, it has been a key observation of recent MRI studies that cerebellar lobules are polymodal structures with segregated, functionally distinct subregions. In fcMRI studies, it is of critical importance to consider these topographical details when placing the seed regions for connectivity analyses. The available fcMRI studies of cerebro-cerebellar connectivity in depression may not have been sufficiently accurate in this regard, potentially explaining the inconsistent cerebellar DMN findings in patients with MDD. Future studies should prefer data-driven extraction of cerebro-cerebellar ICNs. Validation of these findings by means of seed-based connectivity analyses may be performed, if accurate seed placement can be guaranteed based on previous data-driven identification of cerebellar ICN components (17).

Some general limitations of available brain imaging data in depression also apply to cerebellar imaging in MDD patients. First, depression is a clinically and biologically heterogeneous disorder, yet only few brain imaging studies have attempted to subtype patients based on clinical, neuropsychological or neurophysiological features (34). Second, in many studies, heterogeneity of psychotropic medication is a potential confound. Third, there is a lack of longitudinal brain imaging data in depression research. As a consequence, a significant number of fundamental research questions may not be answered. In particular, there is a need to discern the causal influence of depression risk factors on brain morphology and function. These limitations should be addressed when designing future studies to investigate cerebellar contributions to major depression.

This review emphasizes the role of non-motor cerebellar regions in patients with depression. Psychomotor disturbances, particularly psychomotor slowing, can be an important feature of major depression (35). The cerebral correlates of aberrant psychomotor functioning have been subject of several neuroimaging investigations (36–38). It should not be forgotten that future studies of the cerebellum in MDD will clearly benefit from investigating cerebro-cerebellar motor systems.

## Author contributions

MD and RW wrote the manuscript. MS and KK contributed to the interpretation of data.

### Conflict of interest statement

The authors declare that the research was conducted in the absence of any commercial or financial relationships that could be construed as a potential conflict of interest.
